# Herbivores override climate control of grassland production in Yellowstone National Park

**DOI:** 10.1002/ecy.70159

**Published:** 2025-07-20

**Authors:** Douglas A. Frank, Jason D. Fridley

**Affiliations:** ^1^ Department of Biology Syracuse University Syracuse New York USA; ^2^ Department of Biological Sciences Clemson University Clemson South Carolina USA

**Keywords:** aboveground production, climate, grassland, grazing, herbivory, precipitation, temperature, ungulate, Yellowstone National Park

## Abstract

Understanding the factors regulating temporal variation in grassland annual aboveground net primary production (ANPP) is dominated by studying the effects of climate, particularly water, in ungrazed grassland. However, the overwhelming majority of the Earth's grasslands are grazed by large herbivores, which have large effects on ANPP and interact with climate in unknown ways. Here, we analyzed an eight‐year dataset of ANPP across a 26‐year period that included widely variable climatic conditions and consumption rates by herds of elk (*Cervus elaphus*), bison (*Bison bison*), and pronghorn (*Antilocapra americana*) at 25 grassland sites in Yellowstone National Park (YNP). We found that ANPP was primarily a positive function of consumption rate and secondarily affected by a nonlinear temperature effect, with ANPP declining in hot years. Water balance (WB, a measure of soil moisture available to plants) had no significant effect on ANPP. Examining the difference between grazed minus ungrazed (fenced) ANPP (i.e., grazer stimulation) at 13 grassland sites revealed that herbivores increased average ANPP by 20%, with variation across sites and years driven by the amount grazed, temperature, and interactions of temperature with local environment and WB. We found a surprising negative main effect of WB on ANPP stimulation, likely because grazing ameliorated moisture stress in dry years by reducing transpirational moisture loss. Our results demonstrate that Yellowstone grazers override the well‐documented positive effect of moisture on grassland ANPP, which highlights the need to understand how together climate and herbivory regulate production in the world's other grassland ecosystems.

## INTRODUCTION

Between‐year variation in grassland annual aboveground net primary production (ANPP) is usually controlled by current year precipitation (Knapp et al., 2017; Knapp & Smith, 2001; Wilcox et al., 2000) and in some cases the precipitation in previous years (i.e., legacy effect; Sala et al., 2012, Oesterheld et al., 2001, Petrie et al., 2018). Large herbivores also have profound effects on grassland plant and soil processes (Milchunas & Lauenroth, 1993; Zhou et al., 2019). However, determining ANPP in grazed grasslands using methods that account for the material removed by animals has been accomplished in very few grassland ecosystems (Charles et al., 2017; Kleppel & Frank, 2022) and over relatively few years in any single ecosystem. An important yet unresolved question is how climate and grazing together drive temporal variation in grassland processes.

Approximately 25% of Earth's terrestrial surface area is grassland and savanna that is grazed by large herbivores (FAO, 2018; Hufkens et al., 2016; Lai & Kumar, 2020; World Resources Institute, 2000). Thus, determining the factors regulating production in grazed habitats is critical to understanding global biogeochemistry (Schmitz et al., 2014). A number of remote sensing studies have found that climate controls aboveground biomass, usually measured as NDVI, across grazed grassland landscapes (Chi et al., 2018; Li et al., 2021; Reinermann et al., 2020; Yang et al., 2012); however, those studies did not account for the often large percentage of ANPP that was consumed by herbivores. With climatic variation expected to increase (IPCC, 2021), the interaction between climate variation and grazing will likely become more important in the future. Yet, there is a limited understanding of the combined roles of large herbivores and climate in controlling aboveground production, which would be useful in forecasting how changing climatic regimes will impact grazed grasslands worldwide.

Yellowstone National Park has a relatively intact food web and is a powerful system in which to study how natural forces control trophic processes (Turner et al., 2017). Grazing ungulates stimulate grassland production in YNP (Frank et al., 2002, 2016, 2018; Frank & McNaughton, 1993), but how they may interact with climatic conditions to impact forage supply and the capacity for the YNP ecosystem to support its herbivore populations is not clearly understood. In a previous study (Frank, 2007), ANPP at 10 YNP grassland sites was insensitive to a two‐year drought. That analysis only tested for changes in ANPP relative to a year of average moisture, without considering the potentially important effects of variable local drought severity (i.e., local climate conditions), soil chemistry (i.e., nutrient availability), and ungulate consumption rates on ANPP. Theoretically, both climatic conditions and local soil properties should interact with grazing by determining the soil resources (moisture, nutrients) available for plants to regrow after being grazed.

Here, we synthesize eight years of data spanning three decades (1988–1989, 1999–2001, 2012–2014) to examine how variation in local climate, soil chemistry, and grazing intensity affect aboveground production in grasslands of YNP that are grazed by large migratory herds of wild ungulates. In each of the 8 years, aboveground production (in grams per square meter), consumption (in grams per square meter removed) and climatic conditions for sequential sampling intervals during the April to September growing season were determined at 25 grazed grasslands (Appendix S1: Figure S1). In addition, ANPP was calculated for fenced (ungrazed) grassland at 13 of the sites. Pairing grazed and ungrazed grasslands allowed us, for the first time that we are aware, to determine how local climatic conditions, soil chemistry, and ungulate consumption rates control the effect of grazers on ANPP. During the eight study years, water year (November–August) precipitation, a measure of the total moisture available to plants as melted snow and growing‐season precipitation, ranged from the lowest at two weather station sites (Mammoth Hot Springs [MAM], 67% of average; Tower Falls [TF], 67% of average) to the second highest at MAM (121% of average) and third highest at TF (126% of average) during a 40‐year period (1973–2014) (Appendix S1: Figure S2). In addition, the composition of the ungulate community (elk, bison, pronghorn) that grazed YNP grasslands varied among the study periods. Elk were dominant during the first study (1988–1989). Predation and harvests of animals migrating outside YNP during hunting seasons reduced the elk population after gray wolves (*Canis lupus*) were reintroduced in 1995. Then, beginning in 2005, bison numbers began to increase as a result of animals emigrating into the study area from other regions of the park (Geremia et al., 2015). These patterns resulted in total biomass among the three ungulate species in the study system declining monotonically from 5029 to 2607 tonnes during the three studies, with a shift from elk dominance to a slight bison‐dominant population in the last study (Frank et al., 2016). Thus, we were able to examine controls on YNP forage production under a wide range of climatic conditions and grazing pressures.

We addressed three broad hypotheses in three separate analyses. (1) Ungrazed grassland ANPP was controlled by water availability measured as water balance (WB), temperature, site condition, and their interactions. (2) Grazed grassland ANPP was controlled by WB, temperature, site condition, and consumption, and their interactions. (3) The difference between grazed ANPP and ungrazed ANPP (i.e., grazer stimulation of ANPP) at the 13 sites with permanent exclosures was a function of WB, temperature, site condition, and consumption (the amount of plant biomass consumed outside permanent exclosures), and their interactions.

## MATERIALS AND METHODS

Yellowstone National Park is an 8983‐km^2^ preserve in the central Rocky Mountains of the United States, with about 20% occupied by grassland and shrub‐grassland and the remainder by forest and lakes (Despain, 1990). The climate of YNP includes long, cold winters and short, dry summers, with precipitation declining over the growing season and temperatures increasing to a peak in July and then cooling.

### Plant production and consumption measurements

ANPP in grazed grassland and biomass consumed by ungulates were measured using five to six moveable exclosures (1.5 × 1.5 m) at each site. These exclosures were relocated randomly approximately every four weeks at each sampling site during the April to September growing season in each year of the three studies. Aboveground production during the sampling interval was calculated as the difference in shoot biomass in 71 × 71 cm (0.5 m^2^) quadrats in the middle of moveable exclosures between the beginning and end of the sampling interval. Consumption was estimated as the difference between shoot biomass inside and outside moveable exclosures at the end of a sampling interval. Ten to twelve randomly located 71 × 71 cm quadrats were used to sample the grazed grassland. The exception to those methods was that during the 1988–1989 study, biomass was not determined inside moveable exclosures, and zero consumption was recorded when animals were not present at a site during the sampling interval based on wildlife observations, knowledge of the migratory habits of each of the ungulate species, and the lack of any visual evidence of recent grazing at a site.

ANPP of ungrazed grassland was determined inside permanent exclosures. Three randomly located permanent exclosures (15 × 15 m) were used to measure ungrazed production during the 1988–1989 study. Single exclosures were established at each of the study sites during the 1999–2001 (8 × 8 to 10 × 10 m) and 2012–2014 (15 × 15 m) studies. The single exclosure per site in the latter two studies was established in the middle of the site, around which moveable exclosures were placed to sample grazed grassland. Aboveground biomass was measured in four to eight 71 × 71 cm quadrats inside each of the permanent exclosures.

Shoot biomass was estimated with the canopy intersect method that related plant biomass with the number of times a pin inserted in the vegetation at a fixed angle contacted vegetation (Frank & McNaughton, 1990). Shoot biomass at each site was measured in grazed grassland and inside permanent and moveable exclosures on the same days. Grazed and ungrazed ANPP were calculated by summing positive increments of plant biomass inside moveable and permanent exclosures, respectively, during each growing season. The exception was the 1988–1989 study, during which ungrazed ANPP was estimated as the biomass measured during peak standing crop inside the permanent exclosures, a good estimate of ANPP in semiarid grasslands (Lauenroth et al., 1986).

The annual rate of consumption was derived by summing the estimated amount of biomass consumed during each sampling interval. Grazing intensity during the growing season was calculated as the percent of the ANPP that was removed by grazers. Because there was microsite (0.5 m^2^) heterogeneity in plant standing biomass unrelated to herbivory at many study grasslands, there were occasions when consumption was calculated as negative when there was little or no herbivory.

Across the three studies, ungulate consumption and ANPP in grazed grassland were measured at 25 sites (Appendix S1: Figure S1), for two years at 14 sites (10 sites 1988, 1989; four sites 2012, 2013), three years at 10 sites (nine sites 1999–2001, one site 2012–2014), and eight years at one site (1988, 1989, 1999–2001, 2012–2014). Ungrazed grassland was measured inside permanent exclosures at a total of 13 sites (Appendix S1: Figure S2), for two years (1988, 1989) at three sites, three years (1999–2001 or 2012–2014) at nine sites, and eight years at one site (1988, 1988, 1999–2001, 2012–2014).

### Soil and climate variables

Soil C and N content (in percentage) were determined for pooled 0‐ to 10‐cm soil collected at three to five spatially stratified random locations in grazed grassland at each site using a Carlo Erba CNS Analyzer (1988–1989 study) or a CE Elantech Soil Analyzer (1999–2001, 2012–2014 studies). The exception was that for the 1988–1989 study, soil C (in percentage) was calculated from loss on ignition, a reliable estimate of soil C concentration (Hoogsteen et al., 2015).

We used climate data from the PRISM Climate Group (2024) to determine the daily temperature and precipitation during each sampling period during each year at each site. PRISM has been shown to provide accurate high‐resolution climatic estimates in mountainous landscapes (Daly et al., 2017). We obtained PRISM data for each location using the R package *prism* (Hart & Bell, 2015). We derived mean April–September temperature and water balance (WB; precipitation minus potential evapotranspiration, PET) for each year using the “thornthwaite” function in the R package *SPEI* (Bagueria & Vicente‐Serrano, 2023), where PET is calculated according to Thornthwaite (1948).

Including WB permitted us to examine the effect of temperature independent of its influence on soil moisture, but we also tested whether using total growing‐season (April–September) or water‐year (WY, prior November–current August) precipitation resulted in better models.

### Data analysis

All analyses were performed in R 4.3.0 (R Development Core Team, 2023). We used mixed‐effects modeling (R packages *lme4* and *lmerTest*; Bates et al., 2015, Kuznetsova et al., 2017) to examine how site properties, climate, and herbivores influenced grassland ANPP, ungrazed (permanently fenced) ANPP, and the amount that animals stimulated ANPP (grazed ANPP − ungrazed ANPP). We explored interactions because of the potential effects on plant growth by resource availabilities influenced by site condition and climate and the varying demand for those resources by plants grazed at different rates. However, we limited our analyses to two‐way interactions to simplify interpretation of results. We did not have information on the site‐specific abundance of herbivore species, so herbivore composition was not included in the models. All models incorporated spatial and temporal autocorrelation using random intercepts for site and year, respectively. To calculate coefficients of determination for mixed‐effect models, we differentiated between variance explained by fixed effects (marginal *R* ^2^) and that explained by both fixed and random effects (conditional *R* ^2^) using the Nakagawa et al. (2017) method as calculated in the R package *performance* (Lüdecke et al., 2021). We used Z‐scaled covariates to calculate standardized regression coefficients.

Models took the following forms. Ungrazed ANPP (ANPP_*u*_; in grams per square meter per year) was modeled with soil percent C (C_soil_), mean growing‐season (April–September) temperature (*T*_gs_), mean water balance (WB), plus two‐way interactions:

$$\begin{array}{l} {\text{ANPP}}_{u}=\mathrm{WB}+T_{\mathrm{gs}}+{T_{\mathrm{gs}}}^{2}+C_{\text{soil}}+\mathrm{WB}:C_{\text{soil}}\\ \;+C_{\text{soil}}:T_{\mathrm{gs}}+\mathrm{WB}:T_{\mathrm{gs}}+{\mathrm{WB}}_{\mathrm{lag}1}\\ \;+N\left(0,{\sigma^{2}}_{\text{site}}\right)+N\left(0,{\sigma^{2}}_{\text{year}}\right)+N\left(0,{\sigma^{2}}_{\mathrm{res}}\right), \end{array}$$

where normal variances represent site, year, and residual variance, respectively, and WB_lag1_ represents the potential for legacy effects of prior year water supply (Petrie et al., 2018). The analysis of grazed ANPP (ANPP_*g*_) was structured the same, except it included consumption (biomass removed per growing season, CONS; in grams per square meters). We included a second‐order term for *T*_gs_ in all models because of a hump‐backed shaped relationship between *T*_gs_ and ANPP (see *Results*). We used C_soil_ as a proxy for site quality in the ANPP models because of its closer association with ANPP than soil percent N and its dual association with moisture retention and soil nutrient content. Including C_soil_ resulted in greater fit for grazed and ungrazed ANPP models compared to when soil percent N was included. For the grazed ANPP model with many predictors and interactions, we confirmed maximal support for the full model versus all possible submodels using the Akaike information criterion (AIC)‐based “dredge” function of the R package *MuMIn* (Barton, 2024).

Absolute grazing stimulation (STIM; in grams per square meter) and percent (relative to ungrazed ANPP) stimulation were modeled with ANPP_*u*_, CONS, WB, *T*_gs_, and *T*_gs_^2^ as fixed factors and site and year as random factors. ANPP_*u*_ replaced C_soil_ used in previous models as a better estimate of site quality. The model form was as follows:

$$\begin{array}{l} \text{STIM}={\text{ANPP}}_{u}+\mathrm{WB}+T_{\mathrm{gs}}+{T_{\mathrm{gs}}}^{2}+\text{CONS}+{\mathrm{WB}}_{\mathrm{lag}1}\\ \;+{\text{ANPP}}_{u}:T_{\mathrm{gs}}+\text{CONS}:T_{\mathrm{gs}}+{\text{ANPP}}_{u}:\mathrm{WB}\\ \;+\mathrm{WB}:T_{\mathrm{gs}}+\mathrm{WB}:\text{CONS}+{\text{ANPP}}_{u}:\text{CONS}\\ \;+N\left(0,{\sigma^{2}}_{\text{site}}\right)+N\left(0,{\sigma^{2}}_{\text{year}}\right)+N\left(0,{\sigma^{2}}_{\mathrm{res}}\right). \end{array}$$

## RESULTS

ANPP of grazed grassland ranged 26.8–550.8 g m^−2^ year^−1^ among sites and years. Environmental properties varied markedly among the grasslands. Percent soil C and N ranged 2.52%–10.1% and 0.20%–0.94%, respectively (Appendix S1, Table S1) and were highly correlated (*r* = 0.89, *p* ≤ 0.001). Mean growing‐season temperature and WB among sites ranged 7.4–14.9°C and −73 to −32 mm, respectively (Appendix S1: Table S1). The amount of biomass removed during the growing season among years and grasslands ranged 0–265.7 g m^−2^, and mean site values were associated with percent soil C (*r* = 0.45, *p* = 0.004), N (0.37, *p* = 0.023), and mean ANPP (*r* = 0.70, *p* ≤ 0.0001).

**FIGURE 1:**
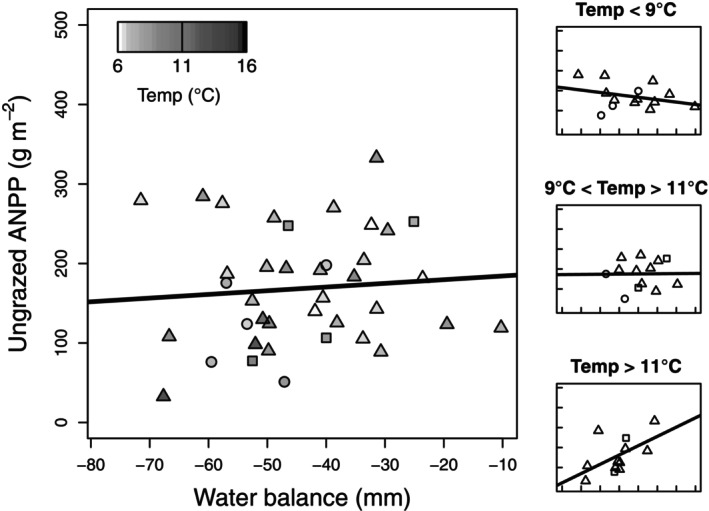
Ungrazed aboveground net primary production (ANPP) on water balance, with ordinary least‐squares regression line (*Y* = 189 + 0.46*X*). Circles, triangles, and squares represent grasslands sampled during 1988–1989, 1999–2001, and 2012–2014, respectively. Shading indicates site growing season mean temperature. Subpanels at right show water balance (WB)–temperature interactions using three equal subsets of the data ranked by temperature.

Modeling ungrazed ANPP inside fences at 13 grasslands revealed a positive interaction between temperature and WB and a positive main effect of WB on ungrazed ANPP (Figure 1, Appendix S1: Table S2). Fixed and random (site, year) factors explained 31% and 64%, respectively, of the total variation in ungrazed ANPP. We found no significant effect of prior‐year WB on ungrazed ANPP (Appendix S1: Table S2). Models using WB explained more variation in ungrazed ANPP than equivalent models using growing‐season or water‐year precipitation (corrected Akaike information criterion [AIC_c_] = 370.9 vs. 372.6 and 373.0, respectively; Appendix S1: Table S3).

**FIGURE 2:**
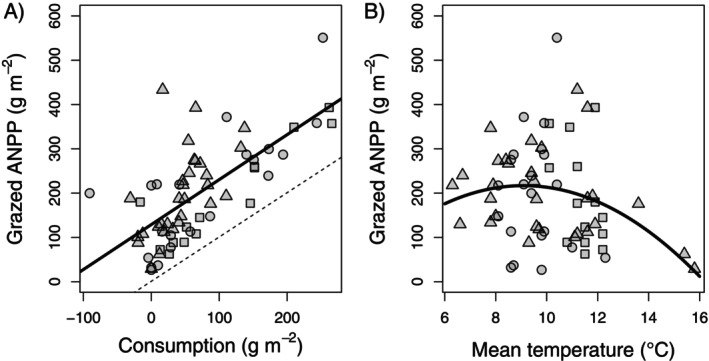
Grazed aboveground net primary production (ANPP) on (A) growing‐season consumption and (B) growing‐season mean temperature. Regression lines are fit via ordinary least squares using (A) linear (*Y* = 129 + 1.01*X*) and (B) quadratic (*Y* = −138 + 78*X* − 4.3*X* ^2^) functions. The dashed line in (A) is a 1:1 relationship indicating the data boundary. Symbol shapes are as in Figure 1.

By contrast, grazed ANPP in YNP grasslands was primarily controlled by a positive effect of consumption (in grams per square meter consumed) and secondarily by a nonlinear, negative second‐order effect of temperature (Figure 2, Appendix S1: Table S4). Fixed and random effects explained 44% and 50% of the variation in grazed ANPP, respectively. Neither WB nor previous‐year WB significantly affected grazed ANPP. As for ungrazed ANPP, using growing‐season or water‐year precipitation in place of WB did not result in better models (Appendix S1: Table S5). In addition, AIC_c_ values for competing submodels of grazed ANPP indicated that the full model of all predictors and two‐way interactions was the model of best data support (Appendix S1: Table S6).

**FIGURE 3:**
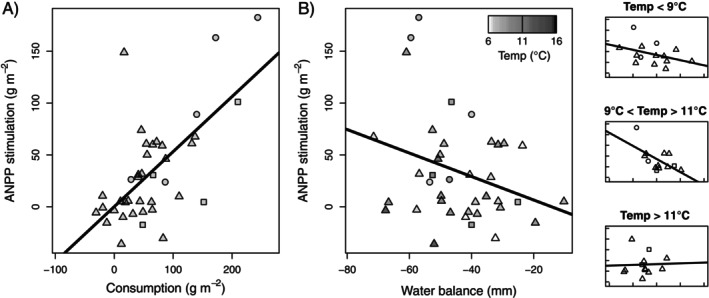
Stimulation of annual aboveground net primary production (ANPP) by herbivores on (A) growing‐season consumption and (B) growing‐season mean water balance with shading representing mean growing‐season temperature. Symbol shapes are as in Figure 1. Ordinary least‐squares regression lines are (A) *Y* = 0.11 + 0.53*X* and (B) *Y* = −16 − 1.13*X*. Subpanels at right show water balance (WB)–temperature interactions using three equal subsets of the data in (B) ranked by temperature.

Comparing ANPP of grazed and ungrazed grassland among sites with permanent fences revealed that herbivores increased ANPP by an average of 20% (mean grazed ANPP = 202.2 g m^−2^ year^−1^; mean ungrazed ANPP = 168.6 g m^−2^ year^−1^), although the amount of ANPP stimulation ranged widely, from −35.9 to +157.1 g m^−2^ year^−1^ across all 13 sites and years. Examining the environmental controls on how much herbivores increased ANPP indicated that consumption rate (in grams per square meter removed per growing season) enhanced stimulation and was the most important predictor in the model (Figure 3A, Appendix S1: Table S7). Temperature had a nonlinear effect on ANPP stimulation, reducing it during relatively hot years (Figure 3B, Appendix S1: Table S7). Temperature interacted with ungrazed ANPP (i.e., site quality) to increase stimulation of ANPP by grazing. WB had a significant negative effect on the stimulation of ANPP by grazing and interacted negatively with temperature to reduce stimulation (Figure 3B, Appendix S1: Table S7). Percent grazer simulation (measured as a percentage of ungrazed ANPP) also was primarily controlled by a positive effect of herbivory (i.e., grazing intensity, percent ANPP consumed; Appendix S1: Table S8).

## DISCUSSION

Our results highlight four principles by which grassland ANPP is regulated in YNP. First, ANPP varies widely across the Yellowstone landscape, ranging by 20‐fold among our grassland study sites, and is associated with gradients of soil percent N and C and the rate of ungulate consumption.

Second, when not grazed, between‐year ANPP is under climate, primarily moisture, control, which is generally consistent with findings from other grassland ecosystems worldwide (Knapp et al., 2017; Knapp & Smith, 2001; Smith et al., 2024; Wilcox et al., 2000). The added explanatory power of including WB, versus measures of precipitation, in models of ungrazed ANPP may be a function of its better estimate of plant moisture availability. The few studies that have examined the role of temperature have found a negative effect on ANPP that is weaker than precipitation (Li et al., 2021; Lin et al., 2014; Mowll et al., 2015). By contrast, in YNP, temperature interacted with WB to increase ANPP in ungrazed grassland, perhaps because of temperature unambiguously reflecting the effects of thermal conditions on plant growth activity, and the relatively cool temperatures that occur the first two months of the growing season in YNP when the bulk of ANPP occurs (Frank & McNaughton, 1992). These results are inconsistent with an earlier finding that ANPP in ungrazed grassland was not affected by drought (Frank, 2007), which may be a function of the larger dataset used here and the analytical framework that determined the effects of temperature, moisture, and local site conditions on ANPP.

Third, climate control of ANPP in ungrazed YNP grasslands was overridden in grazed grassland by a dominant effect of consumption (in grams per square meter removed). The strong impact of herbivores and lack of any influence of climate support the earlier finding that ANPP in grazed YNP grassland is not sensitive to drought.

Fourth, the rate of ANPP stimulation was primarily controlled by the amount of forage consumed by herbivores, similar to its influence on grazed grassland ANPP. However, we were surprised that WB reduced the response of plant growth to grazing (i.e., stimulation) in YNP. This conundrum may be explained by how the removal of transpirational surface area by grazing, and its ameliorating effect on plant moisture stress, interacts with ambient soil moisture conditions. A previous YNP study (Frank et al., 2018) that measured soil moisture over two growing seasons inside and outside permanent exclosures at a dry and mesic grassland showed that grazing increased soil moisture content only when ungrazed soils began to dry in the middle and late growing season. This suggests that the herbivore‐induced reduction in transpirational water loss does not influence soil moisture content when soils are saturated or wet, and it is only during relatively dry conditions that a reduction in transpirational moisture loss has an impact on soil moisture content, and thus can stimulate production. Therefore, stimulation of ANPP by grazers should decline in wet years, but less so in relatively hot years; two patterns that are confirmed in the model results (Figure 3, Appendix S1: Table S7). The negative effect of moisture on stimulation likely nullified the positive effect that WB had on ANPP in ungrazed grasslands.

YNP has been a widely discussed case study exploring the extent to which an increase in predator pressure (wolves, human hunting) triggers a trophic cascade leading to more woody vegetation (aspen, willows) for browsing herbivores (Beschta & Ripple, 2016; Brice et al., 2022). Lost in the discussion has been how YNP grassland, the primary habitat that provides the bulk of forage for Yellowstone's ungulate herds, may or may not conform to trophic theory that would predict a downturn in plant performance with increasing herbivory (Pace et al., 1999). YNP grazers have been shown to increase soil N availability for plants (Frank & Groffman, 1998) and promote an arbuscular mycorrhizal fungal community that is more beneficial to plant hosts than that in ungrazed grasslands (Frank et al., 2003), in addition to increasing soil moisture. Positive feedbacks of herbivory on plants in YNP, and in other grassland ecosystems (Kleppel & Frank, 2022), suggest that trophic theory needs to be re‐evaluated to include grassland ecosystems such as YNP where herbivory can facilitate plant productivity.

Our findings raise two questions. First, how will global change impact forage productivity in YNP? Forecasts for YNP include increasing temperatures, precipitation variability, and drought intensity (USGCRP, 2023). Our results indicating that grazers increase ANPP in relatively dry years suggest that grazers may provide a buffer to drought, which should stabilize the production of forage for YNP herbivores as temperatures warm in the near term. However, we also discovered that hotter years reduced ANPP and the rate at which it was stimulated by herbivores. At what point further increases in temperature and drought reach a tipping point that ends the positive feedbacks of herbivores on their forage production and leads to smaller herbivore herds is not known.

Second, how important is herbivory in regulating ANPP in grasslands other than YNP? The bulk of grazing studies have measured the effects of herbivory on aboveground biomass (Milchunas & Lauenroth, 1993; Zhou et al., 2019), thus ignoring the amount of material consumed and the subsequent compensatory effects of grazing on plant growth. By contrast, studies examining climate effects on grasslands usually measure rates of production (e.g., Knapp et al., 2017; Knapp & Smith, 2001; Smith et al., 2024; Wilcox et al., 2000), which may explain why studying the regulation of grassland production has largely been limited to the role of climate. In the few studies that we are aware of in which production in grazed grassland has been measured while accounting for the material consumed by animals, wild migratory ungulates and livestock altered ANPP by +65% to ˗55% among temperate and tropical grasslands (Bagchi & Ritchie, 2010; Charles et al., 2017; Frank et al., 1998; Irisarri et al., 2016; Knapp et al., 2012; Mipam et al., 2019; Schoenecker et al., 2021; Schönbach et al., 2011). How herbivory interacts with climate in regulating production in other grassland ecosystems needs to be addressed for a more robust and realistic understanding of grassland function and to provide managers with best practices to optimize forage productivity as the climate shifts.

## AUTHOR CONTRIBUTIONS

Douglas A. Frank compiled the dataset and drafted the manuscript. Jason D. Fridley led statistical analyses and commented on the manuscript.

## CONFLICT OF INTEREST STATEMENT

The authors declare no conflicts of interest.

## Supporting information


Appendix S1.

## Data Availability

Data (Frank & Fridley, 2025) are archived on Dryad at https://doi.org/10.5061/dryad.7m0cfxq71.
