# Supplementary material for: Herbivores override climate control of grassland production in Yellowstone National Park
Source: Ecology. 2025 Jul 20;106(7):e70159. doi: 10.1002/ecy.70159 (PMC12277870; doi:10.1002/ecy.70159)
Supplement: Supplementary file 1 — Appendix S1. [file ECY-106-e70159-s001.pdf]

Appendix S1

Authors: Douglas A. Frank and Jason D. Fridley

Article Title: Herbivores override climate control of grassland production in Yellowstone National Park

Journal: Ecology

Figure S1. Map of study sites. Circles, triangles, and squares represent grasslands sampled during 1988-1989, 1999-2001, and 2012-2014, respectively. Solid symbols are locations where ungrazed grassland inside permanent exclosures were studied. Locations of Mammoth Hot Springs (MAM) and Tower Falls (TF) weather stations are denoted with stars.

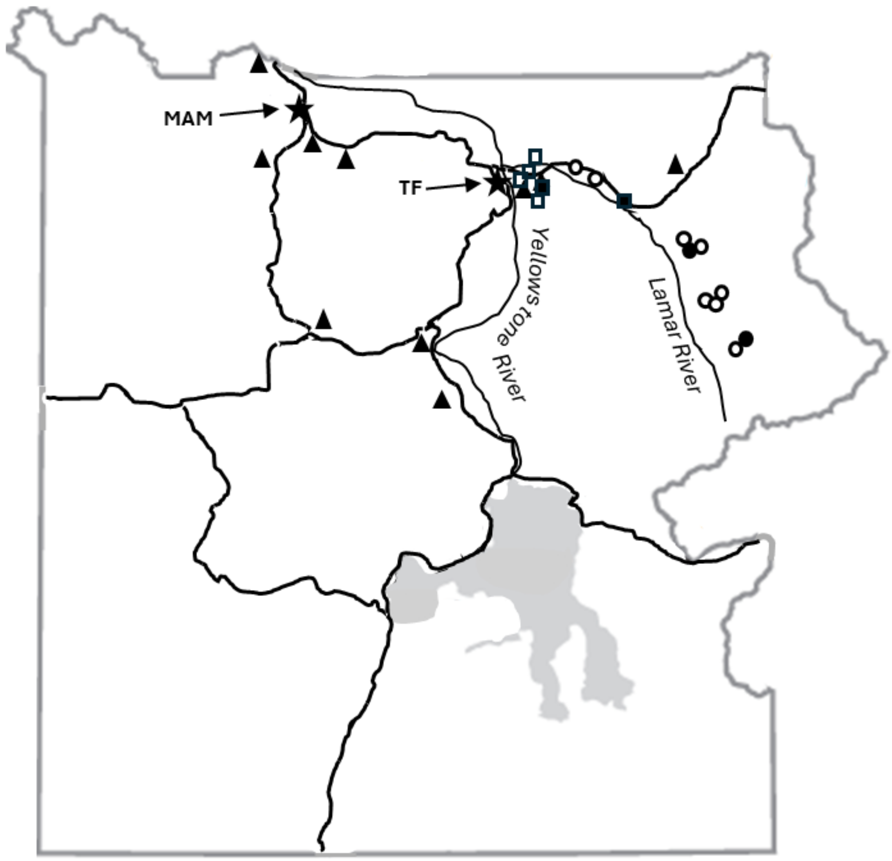

Figure S2. Water year (November – August) precipitation for 1970-2014 at two park weather stations that provided the most complete precipitation records for the period. MAM = Mammoth Hot Springs; TF = Tower Falls. Shaded areas are years that studies were conducted.

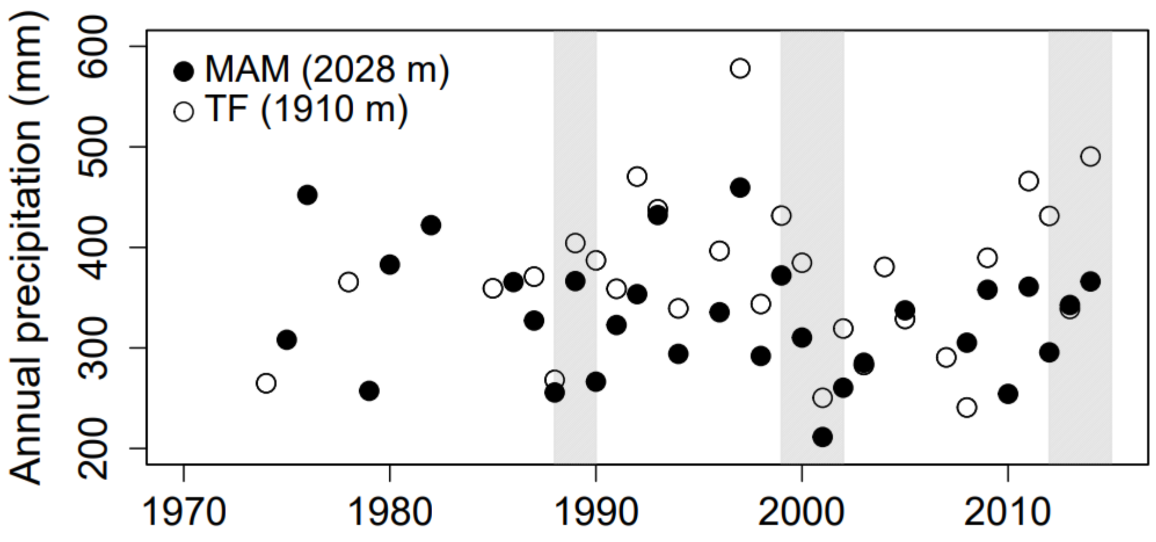

Table S1. Site properties.

Site ID	elevation (m)	Soil C (%)	Soil N (%)	Mean growing season temperature	Mean water balance	Grazed ANPP	Ungrazed ANPP	Growing season consumption
cb	1909	2.52	0.22	11.37	-49.41	97.3	98.9	30.6
open forest	2428	4.42	0.2	8.75	-49.04	208.5	-	-45.4
l1	1998	7.76	0.5	9.75	-49.57	461.3	-	181.7
l2	1999	3.30	0.2	9.75	-53.70	219.6	-	24.7
lcr	2342	7.57	0.7	9.3	-32.74	75.1	-	12.8
lsg	2294	8.54	0.7	9.9	-72.99	286.9	-	194.0
rbm	2428	8.12	0.6	8.75	-56.48	193.5	100.1	129.3
swale	2318	10.07	0.7	9.3	-48.48	322.6	186.8	191.8
ucm	2355	9.90	0.8	9.2	-64.50	287.3	-	161.9
ucr	2575	5.27	0.1	9.2	-61.79	29.4	-	0.0
usg	2450	6.64	0.5	9.2	-61.84	108.9	-	43.1
lburn	2294	8.54	0.7	9.1	-34.78	208.7	-	141.5
lsgb	2294	8.54	0.7	8.7	-36.20	122.1	-	0.0
black	2072	3.20	0.29	10.53	-33.32	98.9	100.9	-17.3
c	2423	7.43	0.58	7.5	-43.37	165.4	156.5	35.1
gh	2267	5.50	0.45	8.97	-32.19	161.6	125.8	32.4
hv	2377	6.12	0.52	7.4	-53.82	279.2	267.8	94.9
lvbench	2028	8.38	0.83	7.87	-31.97	260.6	218.6	72.2
mam	2028	9.88	0.73	11.07	-37.19	227.9	206.2	70.3
n	2286	9.23	0.94	8.93	-43.88	206.5	181.3	48.5
sc	1661	2.52	0.23	14.93	-56.82	89.2	86.9	33.1
sr	1914	6.20	0.51	10.73	-47.09	381.4	291.6	45.5
becca	2023	7.80	0.5	10.37	-42.42	321.0	250.1	209.2
casp	1926	5.64	0.42	11.85	-49.47	134.2	-	61.5
pereg	1889	4.63	0.4	11.55	-50.05	219.9	-	67.6
ssr	1922	4.72	0.42	11.55	-56.32	285.1	-	203.7
wden	1890	3.42	0.29	11.85	-53.91	67.5	-	19.8

Table S2. Results for the ungrazed grassland ANPP model. (A) Factors are water balance (WB), previous-year water balance (WB_{lag1}), percent soil C (Soil C), and mean growing season temperature (Temp). Values are estimate (Est.), standard error (SE), degrees of freedom (df), t value (t), and probability value (p). (B) Variances explained by conditional and marginal effects.

A.

Factor	Est.	SE	df	t	p
Intercept	161.1	24.61	11.82	6.55	<0.001
WB	22.53	9.07	20.48	2.48	0.02
Soil C	24.09	20.47	11.76	1.18	0.26
WB_{lag}	12.62	15.85	12.27	0.8	0.44
Temp	8.61	20.29	11.46	0.42	0.68
$Temp^2$	-6.85	5.3	20.53	-1.29	0.21
WB:Soil C	3.43	8.45	18.25	0.41	0.69
Temp:Soil C	-3.01	10.13	17.43	-0.3	0.77
WB:Temp	18.3	6.43	16.1	2.85	0.01

B.

Conditional R^2	0.954
Marginal R^2	0.310

Table S3. Ungrazed ANPP models using (A) growing-season (April-August; GSP) or (B) water-year (November-April; WYP) precipitation instead of water balance. Columns are as in Table S2. AIC values of models using WB, GSP, and WYP variables were 370.9, 372.6, and 373.0, supporting WB as the best water supply variable.

A.

Factor	Est.	SE	df	t	p
Intercept	187.46	20.67	12.04	9.07	<0.001
GSP	18.02	12.79	28.56	1.41	0.17
Soil C	15.43	19.69	12.22	0.78	0.45
GSP_{lag}	7.2	10.61	24.88	0.68	0.5
Temp	21.32	16.67	29.4	1.28	0.21
$Temp^2$	-7.04	11.56	24.65	-0.61	0.55
GSP:Soil C	20.39	12.46	28.8	1.64	0.11
Temp:Soil C	18.15	15.33	29.22	1.18	0.25
GSP:Temp	8.98	12.62	24.47	0.71	0.48

B.

Factor	Est.	SE	df	t	p
Intercept	171.41	25.37	9.01	6.76	<0.001
WYP	-29.11	39.13	18.69	-0.74	0.47
Soil C	23.53	17.85	9.12	1.32	0.22
WYP_{lag}	-19.13	17.03	15.88	-1.12	0.28
Temp	-33.44	30.15	20.26	-1.11	0.28
$Temp^2$	-19.08	7.85	18.05	-2.43	0.03
WYP:Soil C	-17.75	18	21.17	-0.99	0.34
Temp:Soil C	-15.09	16.98	21.49	-0.89	0.38
WYP:Temp	-12.26	12.48	26.6	-0.98	0.33

Table S4. Results for the grazed grassland ANPP model. (A) Factors, values, and (B) variances are as Table S2, with exception that the factor total growing season consumption (Consump) has been added.

A.

Factor	Est.	SE	df	t	p
(Intercept)	215.35	20.31	24.85	10.6	<0.001
WB	-1.37	8.18	38.24	-0.17	0.87
Soil C	7.55	16.27	47.15	0.46	0.64
Temp	13.91	19.22	13.44	0.72	0.48
$Temp^2$	-17.12	7.11	36.74	-2.41	0.02
Consump	63.11	11.21	39.1	5.63	<0.001
WB_{lag}	20.54	15.87	9.66	1.29	0.23
WB:Soil C	-13.68	10.77	39.43	-1.27	0.21
Temp:Soil C	-8.95	11.98	40.56	-0.75	0.46
WB:Temp	0.36	7.88	31.43	0.05	0.96
WB:Consump	6.09	9.96	32.86	0.61	0.55
Soil C:Consump	-4.93	9.04	33.45	-0.55	0.59
Temp:Consump	0.71	11.7	35.68	0.06	0.95

B.

Conditional R^2	0.940
Marginal R^2	0.438

Table S5. Grazed ANPP modeled with (A) growing season precipitation (GSP) and (B) water year precipitation (WYP) in place of water balance. Other factors and all values are as Table S4. AIC values of models using WB, GSP, and WYP variables were 721.5, 723.0, and 721.7, resp.

A.

Factor	Est.	SE	df	t	p
(Intercept)	216.85	20.4	22.95	10.63	<0.001
GSP	-16.34	15.71	35.35	-1.04	0.31
Soil C	10.31	15.96	45.4	0.65	0.52
Temp	-2.24	23.5	31.68	-0.1	0.92
$Temp^2$	-22.48	12.16	37.51	-1.85	0.07
Consump	62.41	10.34	38.13	6.03	<0.001
GSP_{lag}	8	15.89	13.31	0.5	0.62
GSP:Soil C	-20.42	14.33	42.75	-1.42	0.16
Temp:Soil C	-13.09	15.72	45.9	-0.83	0.41
GSP:Temp	-7.48	12.91	40.64	-0.58	0.57
GSP:Consump	11.73	10.24	34.08	1.15	0.26
Soil C:Consump	-3.63	9.1	34.63	-0.4	0.69
Temp:Consump	3.77	11.93	35.52	0.32	0.75

B.

Factor	Est.	SE	df	t	p
(Intercept)	208.58	19.65	23.62	10.62	<0.001
WYP	-28.55	28.44	6.75	-1	0.35
Soil C	12.49	15.65	47.16	0.8	0.43
Temp	-12.75	26.81	14.29	-0.48	0.64
$Temp^2$	-17.87	9.95	36.42	-1.8	0.08
Consump	64.49	11.25	38.58	5.73	<0.001
WYP_{lag}	7.69	15.36	16.92	0.5	0.62
WYP:Soil C	-25.59	18.69	34.66	-1.37	0.18
Temp:Soil C	-15.85	18.88	37.98	-0.84	0.41
WYP:Temp	-11.63	10.61	16.83	-1.1	0.29
WYP:Consump	21.23	14.19	31.87	1.5	0.14
Soil C:Consump	0	9.19	38.05	0	1.00
Temp:Consump	14.22	14.02	35.11	1.01	0.32

Table S6. Coefficients and goodness-of-fit criteria of the top 10 best-fit models of grazed ANPP, ranked by Akaike weight. The maximum model of all predictors and two-way interactions is supported as the best model in comparison with all submodels.

Intercept	Consump	Temp	Temp2	WB lag1	Soil C	WB	Temp: Consump	Consump: Soil C	WB: Consump	Temp: Soil C	WB: Temp	WB: Soil C	df	logLik	AICc	delta	Akaike weight
215.35	63.11	13.91	-17.12	20.54	7.55	-1.37	0.71	-4.93	6.09	-8.95	0.36	-13.68	16	-304.10	651.54	0.00	0.32
215.46	63.18	13.95	-17.40	20.88	7.14	-1.46	0.70	-5.05	6.25	-9.38		-14.06	15	-307.08	653.96	2.42	0.10
213.13	60.42	14.33	-16.50	18.86	9.74	-1.01	3.37		5.57	-8.14	1.16	-12.82	15	-307.37	654.54	3.00	0.07
215.56	63.44	13.83	-17.32	20.88	7.10	-1.35		-5.21	6.16	-9.13	0.28	-13.88	15	-307.48	654.75	3.21	0.06
213.56	59.84	14.55	-16.39	20.21	8.57	-0.42	1.47	-4.43		-8.34	2.07	-8.65	15	-307.51	654.81	3.27	0.06
216.53	63.50	15.28	-14.99	18.07	10.47	-0.92	-0.32	-4.02	5.38		1.28	-10.49	15	-307.76	655.31	3.76	0.05
212.85	58.23	15.82	-14.09	17.54	13.65	1.47	2.43	-3.17	-3.44	-2.17	5.32		15	-308.18	656.15	4.60	0.03
211.96	62.10	3.69	-13.79		10.63	-1.69	2.62	-2.38	5.32	-5.36	1.59	-11.67	15	-308.60	656.99	5.45	0.02
213.50	60.66	14.40	-17.20	19.28	9.16	-1.41	3.34		6.08	-8.71		-13.73	14	-310.33	657.07	5.52	0.02
215.63	63.49	13.87	-17.55	21.21	6.72	-1.41		-5.31	6.28	-9.54		-14.21	14	-310.43	657.26	5.72	0.02

Table S7. Model results for the amount that herbivores stimulated ANPP ($gm^{-2} yr^{-1}$). (A) Factors, values, and (B) variances are as Table S4, with exception that the factor, ANPP in paired ungrazed grassland (UngANPP), has been included in place of Soil C.

A.

	Est.	SE	df	t	P
(Intercept)	51.72	15.53	22.56	3.33	<0.001
UngANPP	4.84	6.95	10.47	0.70	0.50
WB	-24.58	5.96	21.28	-4.12	<0.001
WB_{lag}	-0.10	0.47	24.93	-0.22	0.83
Temp	8.73	6.58	22.18	1.33	0.20
Consump	43.99	7.14	18.65	6.16	<0.001
$Temp^2$	-8.40	4.03	22.70	-2.08	0.05
WB:Temp	-23.50	6.55	24.30	-3.59	<0.001
Temp:UngANPP	17.26	6.70	25.89	2.58	0.02
Temp:Consump	-14.77	9.42	24.63	-1.57	0.13
WB:UngANPP	-6.54	6.08	20.50	-1.08	0.29
WB:Consump	-12.83	9.01	23.59	-1.42	0.17
UngANPP: Consump	-10.60	7.71	25.43	-1.37	0.18

B.

Conditional R^2	0.805
Marginal R^2	0.725

Table S8. Model results for the percentage that herbivores stimulated ANPP ($gm^{-2}yr^{-1}$ increased/ ungrazed ANPP). (A) Factors, values, and (B) variances are as Table S7, with exception that the factor, the percentage of ANPP consumed (100*[consump/ grazed ANPP]) is included.

A.

	Est.	SE	df	t	P
(Intercept)	6.13	1.37	8.69	4.47	<0.001
UngANPP	-0.14	0.49	3.28	-0.29	0.79
WB	-0.42	0.41	7.41	-1.03	0.34
WB_{lag}	0.07	0.04	7.18	1.64	0.14
Temp	0.21	0.42	6.48	0.49	0.64
%Consumed	1.64	0.48	5.85	3.44	0.01
$Temp^2$	0.00	0.22	8.87	0.02	0.98
WB:Temp	-0.65	0.42	6.53	-1.54	0.17
Temp:UngANPP	0.54	0.46	7.17	1.17	0.28
Temp:%Consumed	-0.95	0.55	8.37	-1.71	0.12
WB:UngANPP	-0.39	0.34	8.83	-1.15	0.28
WB:%Consumed	-0.55	0.51	8.65	-1.08	0.31
UngANPP:%Consumed	-0.75	0.46	4.18	-1.63	0.18

B.

Conditional R^2	0.719
Marginal R^2	0.584
